# Tuberculous sacroiliitis with secondary psoas abscess in an older patient: a case report

**DOI:** 10.1186/s13256-018-1754-4

**Published:** 2018-08-18

**Authors:** Luisa Kramer, Vanessa Geib, John Evison, Ekkehardt Altpeter, Jasmin Basedow, Jan Brügger

**Affiliations:** 1 Department of Internal Medicine, Sonnenhof Hospital, Buchserstrasse 30, 3006 Bern, Switzerland; 2 Department of Infectious Disease, Lindenhof Hospital, Bremgartenstrasse 117, 3001 Bern, Switzerland; 3 Federal Office of Public Health, Schwarzenburgstrasse 157, 3003 Bern, Switzerland; 4 Department of Radiology, Sonnenhof Hospital, Buchserstrasse 30, 3006 Bern, Switzerland

**Keywords:** Tuberculosis, Psoas abscess, Sacroiliac joint, Sacroiliitis, Infection

## Abstract

**Background:**

Tuberculosis is the leading infectious cause of death worldwide. Among native Swiss people, tuberculosis is more common in older people than in younger people. Approximately 25–30% of reported cases of tuberculosis are purely extrapulmonary; skeletal tuberculosis is reported in 3–5% of cases. The purpose of this case report is to draw attention to the diagnostic challenge of tuberculous sacroiliitis with secondary psoas abscess, as this clinical picture is very rare.

**Case presentation:**

A magnetic resonance imaging scan of an 85-year-old (Swiss-German) Caucasian woman with chronic left-sided hip pain and limitation of hip joint movement showed a progressive destruction of her sacroiliac joint and a large collection in her left iliopsoas muscle. Drainage of the abscess revealed pus; a polymerase chain reaction assay was positive for *Mycobacterium tuberculosis* complex. Tuberculous sacroiliitis with secondary iliopsoas abscess was diagnosed 9 months after the start of the symptoms. Combination treatment with isoniazid, rifampicin, pyrazinamide, and ethambutol was started.

**Conclusions:**

Sacroiliitis with secondary psoas abscess is an unusual cause of hip pain and is likely to be overlooked since joint pain of the lower extremity and lumbar pain are some of the most common complaints in older individuals. A high level of suspicion and invasive diagnostic procedures are needed for timely diagnosis of tuberculous sacroiliitis not only in immunocompromised patients living in or originating from endemic areas.

## Background

Tuberculosis (TB) remains one of the most common infectious diseases in the world, affecting more than 10 million people each year. In 2015, it was one of the top ten causes of death worldwide, ranking above human immunodeficiency virus (HIV) infection and acquired immune deficiency syndrome (AIDS). The incidence and prevalence of TB have been declining globally since the early 1990s; the TB mortality rate (per 100,000 population) fell by 37% between 2000 and 2016 [1].

TB preferentially affects immunocompromised patients or those living in or originating from endemic areas. In Switzerland, approximately 600 cases of TB are diagnosed per year, frequently in young immigrants. From 2011 to 2016, 75–80% of patients with TB were of foreign origin. The median age of all patients of Swiss origin with TB for each of these years was between 62 and 70 years.

The lung, as the portal of entry, is affected by TB in more than two-thirds of all cases. The skeletal system is involved in approximately 3–5% of cases. Sacroiliac joint involvement is rare but exact numbers are unavailable. As a complication of skeletal TB (most commonly seen in vertebral osteomyelitis), a psoas abscess can develop which produces nonspecific symptoms.

There are two types of psoas abscess. In the primary type, more common in tropical areas, no underlying focus of infection is identified. It is believed to be caused by unrecognized staphylococcal bacteremia. Secondary psoas abscesses are more common in temperate regions. They result from the extension of intraabdominal or vertebral infections such as appendicitis, diverticulitis, Crohn’s disease, or vertebral osteomyelitis. Fever, abdominal or back pain, and limitation of hip joint movement comprise the classic triad of psoas abscess, but less than 50% of all patients present with these symptoms [2, 3]. The nonspecific presentation and lack of awareness of psoas abscess lead to diagnostic delay and increased morbidity [4].

Sacroiliac joint TB with secondary psoas abscess is very rare and is characterized by slow progression. The radiological features of sacroiliitis seen in conventional X-rays require several months to develop (for example, erosion of the joints, loss of cortical margins, virtual joint space, or fusion of the joint). Bone marrow edema is an early sign of sacroiliitis detected by magnetic resonance imaging (MRI). A joint effusion can be an early MRI sign with high predictive value. With the increased use of computed tomography (CT) scans to evaluate patients with unknown foci of sepsis or chronic pain, psoas abscesses are diagnosed and reported more frequently [5].

Psoas abscesses detected by imaging studies need to be aspirated and/or biopsied for further diagnostic microbiologic evaluation. The recommended treatment of a pyogenic psoas abscess includes antimicrobial therapy combined with percutaneous drainage [6]. Tuberculous psoas abscesses, however, often heal with antituberculous drugs and without drainage. Some experts recommend 9 months of treatment for TB of bones or joints because of the difficulties of assessing treatment response [7].

The presented case demonstrates how variegated TB can present in clinical practice and that the rarity and nonspecific clinical manifestation of infectious sacroiliitis caused by TB often lead to delayed proper diagnosis. In the current literature, only a few cases describe the association of TB and infectious sacroiliitis, hence the clinician or first responder needs to keep this differential diagnosis in mind when approaching a patient with common back pain.

## Case presentation

**Fig. 1:**
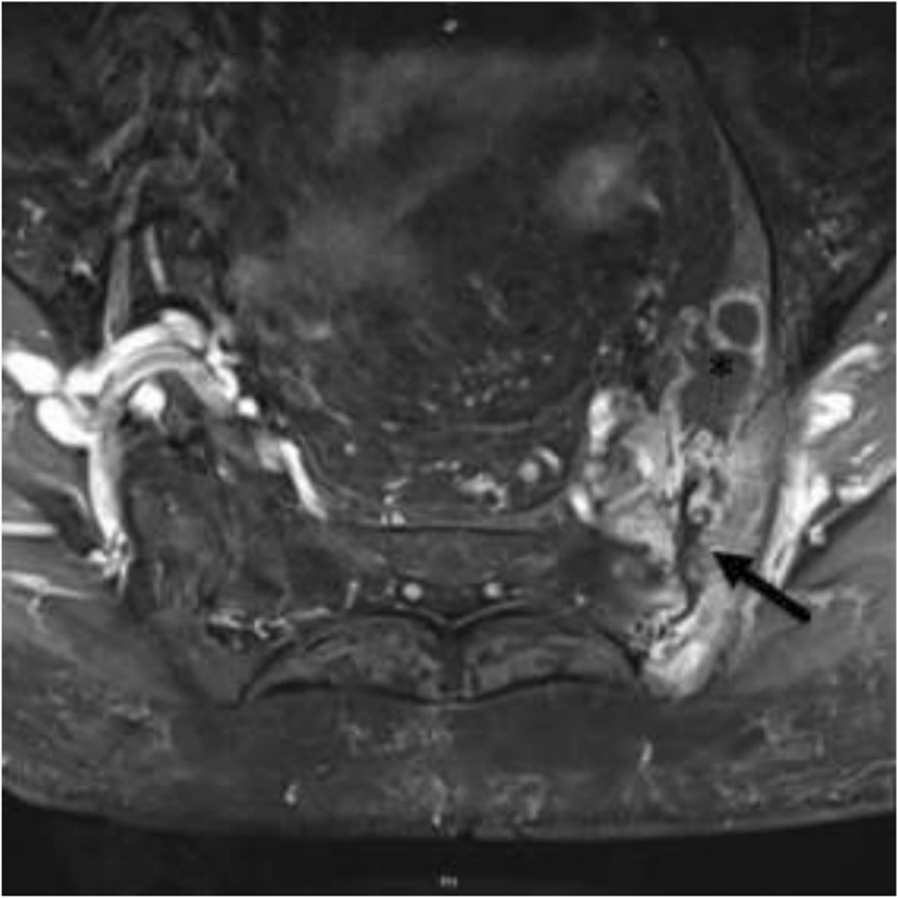
Abdominal magnetic resonance image showing destruction of the left iliac joint with surrounding edema (arrow) and a small collection of fluid ventral to the iliosacral joint (star)

**Fig. 2:**
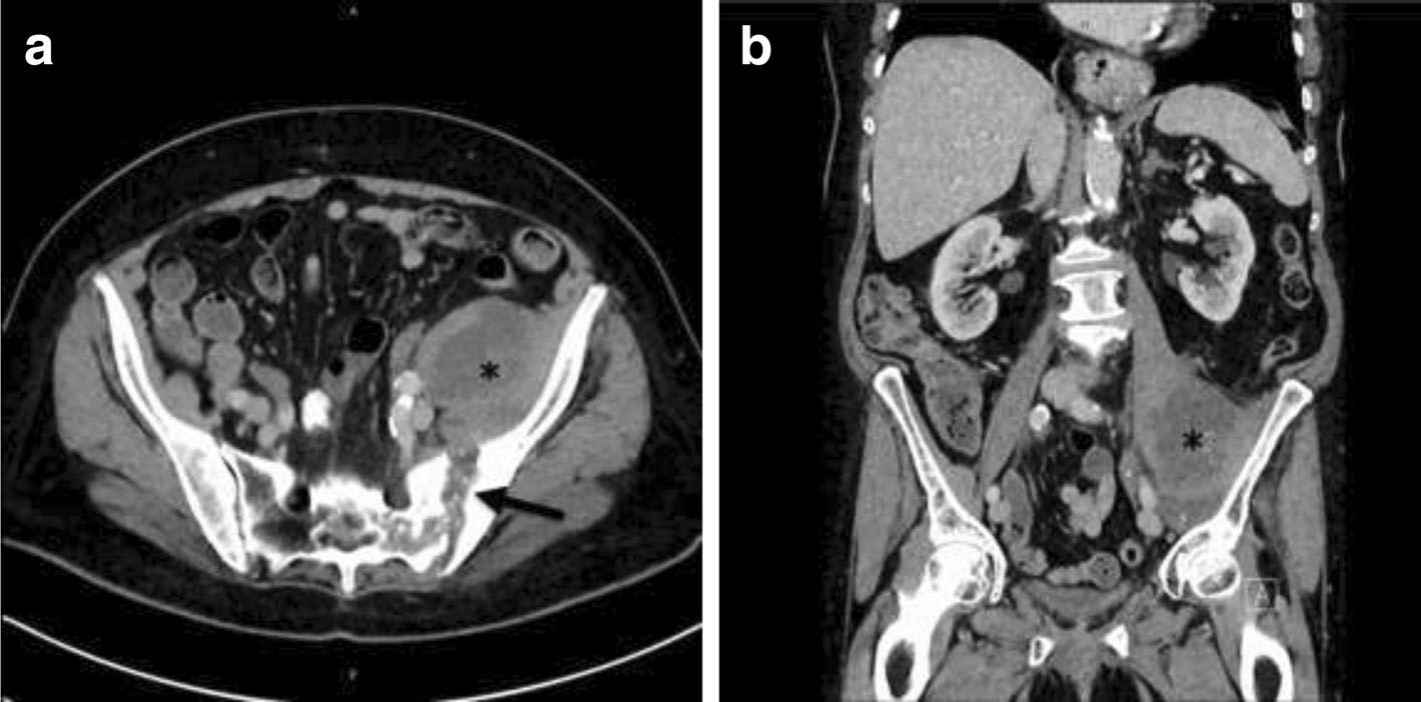
Abdominal computed tomography images showing left psoas abscess (*star*) and destruction of the sacroiliac joint (*arrow*). **a** Coronal image; **b** axial image

An 85-year-old (Swiss-German) Caucasian woman, born and raised in Switzerland, presented to her family doctor with left hip pain and limitation of hip joint movement but no fever, weight loss, or night sweats. Her medical history included hypertension, hypothyroidism, and asthma. Her daily medication was valsartan, levothyroxine, and ciclesonide oral Inhalation. Her family history was uneventful. The widowed patient lived on her own; she did not smoke tobacco or drink alcohol. An MRI scan showed advanced degeneration of her lumbar spine and destruction of the left iliac joint with surrounding edema and a small collection of fluid ventral to the iliosacral joint (see Fig. 1). Arthritis of her left sacroiliac joint was diagnosed. Despite numerous sacroiliac joint infiltrations and thermocoagulation of sensory nerves there was no long-lasting improvement. Her exacerbated lumbar pain led to repeated CT and MRI scans 9 months after the onset of the lumbar pain. Imaging showed progressive destruction of the sacroiliac joint and a 10 × 8 × 7 cm fluid collection in her left iliopsoas muscle (Fig. 2).

She was in good health except for her persevering hip pain. An examination revealed normal vital signs and no fever. Sensations were normal in both lower limbs. Muscle strength was grade M5 throughout (Medical Research Council scale of muscle strength), and deep tendon reflexes were normal. Laboratory testing showed low hemoglobin (100 g/l, reference range 118–158 g/l), peripheral monocytosis with a normal leukocyte count, and a C-reactive protein (CRP) level of 20 mg/l (reference < 5 mg/l). Her liver and kidney function were sufficient, the electrolytes were normal. No antibiotics were used before abscess drainage. After the abscess was drained surgically, her condition improved, and she remained afebrile. Blood cultures remained negative. A presumptive diagnosis of a pyogenic abscess secondary to a bacterial sacroiliitis after numerous infiltrations was made.

Seven biopsies from the pus-like fluid were collected and analyzed. Leukocytes were present in the Gram-stain smear; acid-fast bacilli could be visualized in auramine-rhodamine stain. The aerobic and anaerobic cultures showed no bacterial growth, neither did the enrichment cultures. A polymerase chain reaction (PCR) assay for TB was positive. *Mycobacterium tuberculosis* complex cultures were found to be positive. Drug susceptibility testing revealed a *M*. *tuberculosis* isolate fully susceptible to all first-line drugs. An X-ray film of her thorax was normal, and acid-fast bacteria staining of sputum was negative. Therefore, tuberculous sacroiliitis with secondary iliopsoas abscess was diagnosed and combination treatment with isoniazid (100 mg), rifampicin (240 mg), pyrazinamide (600 mg), and ethambutol (400 mg) was started. The potency of the therapy was increased over the following days (isoniazid (250 mg), rifampicin (600 mg), pyrazinamide (1500 mg), and ethambutol (1000 mg).

For further observation of the treatment and rehabilitation our patient was referred to a rehabilitation hospital where she developed a hypoactive delirium. After exclusion of all other causes the delirium had to be attributed to the antituberculous drugs. Laboratory testing showed persistent low hemoglobin (109 g/l) and a CRP level of 10 mg/l. Her liver enzymes were elevated: alanine aminotransferase (ALT) 183 U/l (reference range < 35 U/l) and aspartate aminotransferase (AST) 149 U/l (reference range < 35 U/l). Her kidney function and the electrolytes were normal. Despite discontinuation of pyrazinamide and ethambutol her hypoactive delirium did not resolve. Therefore her antituberculous treatment was discontinued and she started to improve. After a treatment interruption of 10 days an alternative treatment with rifampicin and moxifloxacin was started.

A control imaging 9 months after therapy initiation showed persistent destruction of her left iliac joint with regressed edema and fluid collections. Repeated laboratory tests showed normal leukocytes, CRP, and liver enzymes, only her erythrocyte sedimentation rate was still elevated (40 mm/hour, reference range < 12 mm/hour). Her condition showed continuous improvement; she now lives on her own with nursing assistance once a week. She has hardly any pain and she has no deficits in sensorimotor function. After a total of 12 months the antibacterial therapy with rifampicin and moxifloxacin was discontinued; a control MRI is planned.

## Discussion

The TB incidence rate continues to fall globally but TB remains a major global health problem. Reports of native Swiss people with the disease have continued to decline over the last years. Physicians’ awareness of and experience with TB has decreased because of the low incidence of the disease (624 cases of TB in Switzerland in 2016). TB in older Swiss individuals reflects widespread infection with *M. tuberculosis* when they were children and an increased risk of disease reactivation when the immune system weakens in old age.

The hallmark of bone and joint TB is the predominance of local symptoms over systemic symptoms, as demonstrated in this case. Pain is the most common complaint. Therefore, it is important to consider skeletal TB in older patients with chronic back or hip pain. Extrapulmonary TB and particularly TB of the bones, joints, and spine are very rare diseases with an annual case load of 19 to 36 cases in Switzerland (Table 1). Diagnosis of bone and joint TB relies on culture of biopsy material from the affected areas; sputum is negative except for the very few cases with concomitant pulmonary TB. The main species identified is *M*. *tuberculosis*. Multidrug-resistant TB (MDR-TB) is rare in these patients.

**Table 1:** Tuberculosis of bone/joints and spine in Switzerland, 2011 to 2016

Year of disease onset	2011	2012	2013	2014	2015	2016
All forms of tuberculosis	589	485	540	495	568	624
Pulmonary	300	255	284	262	280	300
Extrapulmonary	180	141	151	145	152	184
Pulmonary and extrapulmonary	109	89	105	88	136	140
Total bone/joints and spine	36	25	28	21	19	23
Site of disease						
Extrapulmonary	28	20	17	17	14	12
Pulmonary and extrapulmonary	8	5	11	4	5	11
Microbiology						
Sputum positive	1	0	2	1	0	2
Culture positive	30	22	26	19	19	22
MDR-TB: Resistance to isoniazid and rifampicin	1	0	1	1	0	1
Origin						
Swiss	6	3	5	3	4	3
Foreign	30	22	23	17	15	20
Unknown	0	0	0	1	0	0
Age in years						
Median	44	36	45	42	36	32
Range	1 to 89	21 to 85	8 to 84	22 to 89	19 to 89	22 to 91
Sex						
Male	17	14	21	11	8	13
Female	19	11	7	10	11	10

*MDR-TB* multidrug-resistant tuberculosis

When evaluating back and hip pain, red flags include weight loss, no change in pain status after treatment, pain at night or at rest, and neurological symptoms. A low level of inflammatory reactions and degenerative changes normally seen in older individuals may mask the radiographic features of tuberculous spondylitis or sacroiliitis [8].

This case report describes an 85-year-old (Swiss-German) Caucasian woman with persistent hip pain for 9 months. A lumbar spine MRI showed fluid collections in her left iliopsoas muscle; after drainage and analysis of the pus-like fluid, tuberculous sacroiliitis with secondary iliopsoas abscess was diagnosed. Skeletal TB with secondary abscess is most commonly seen in vertebral osteomyelitis, the sacroiliac joint is rarely affected. Previous case reports of tuberculous sacroiliitis complicated by a psoas abscess are very rare [9–11]. Due to the rarity of this condition and atypical presentation, diagnosis remains a challenge and delayed diagnosis or even wrong treatment is possible. This case highlights the need for vigilance in the older patient population where skeletal pain is one of the most common complaints. As described in this report, patients who are not immunocompromised and who do not live in or originate from endemic areas can also be affected.

## Conclusions

Chronic low back or hip pain should raise suspicion of TB of the spine, the ilium, or the sacroiliac joint, possibly complicated by a psoas abscess. Chronic skeletal pain with fever, weight loss, and other nonspecific symptoms should be carefully assessed in Western countries as they may be symptoms of TB. Lumbar pain and joint pain of the lower extremity are common complaints of older individuals, who are also at increased risk of reactivated TB.

This case report documents TB as an unusual etiology of hip pain. Our case confirms that the rarity and nonspecific clinical manifestation of infectious sacroiliitis usually lead to delayed diagnosis. Early diagnosis and appropriate management remain a challenge. A high degree of suspicion, imaging studies, and invasive diagnostic procedures may be necessary.

## Abbreviations

AIDS: Acquired immune deficiency syndrome
ALT: Alanine aminotransferase
AST: Aspartate aminotransferase
CRP: C-reactive protein
CT: Computed tomography
HIV: Human immunodeficiency virus
MDR-TB: Multidrug-resistant tuberculosis
MRI: Magnetic resonance imaging
PCR: Polymerase chain reaction
TB: Tuberculosis
